# Biomarkers to guide the use of antibiotics for acute exacerbations of COPD (AECOPD): a systematic review and meta-analysis

**DOI:** 10.1186/s12890-022-01958-4

**Published:** 2022-05-13

**Authors:** George Hoult, David Gillespie, Tom M. A. Wilkinson, Mike Thomas, Nick A. Francis

**Affiliations:** 1 Cardiff University School of Medicine, UHW Main Building, Heath Park, Cardiff, CF14 4XN UK; 2 Nuffield Department of Primary Care Health Sciences, University of Oxford, Oxford, UK; 3 Centre for Trials Research, College of Biomedical and Life Sciences, Cardiff University, Cardiff, UK; 4 Clinical and Experimental Sciences, Faculty of Medicine, Southampton General Hospital, Southampton University, Mailpoint 810, Level F, South Block, Southampton, SO16 6YD UK; 5 NIHR Southampton BRC – Respiratory Theme, Faculty of Medicine, Southampton General Hospital, Southampton University, Mailpoint 810, Level F, South Block, Southampton, SO16 6YD UK; 6 Primary Care Research Centre, School of Primary Care, Population Sciences and Medical Education, Aldermoor Health Centre, Faculty of Medicine, University of Southampton, Aldermoor Close, Southampton, SO16 5ST UK

**Keywords:** COPD, Exacerbation, Antibiotics, Infection, Biomarkers, Systematic review

## Abstract

**Background:**

Antibiotics are frequently prescribed for acute exacerbations of COPD (AECOPD) even though most do not have a bacterial aetiology. Biomarkers may help clinicians target antibiotic use by identifying AECOPD caused by bacterial pathogens. We aimed to summarise current evidence on the diagnostic accuracy of biomarkers for detecting bacterial versus non-bacterial AECOPD.

**Methods:**

We searched Embase and Medline using a search strategy including terms for COPD, biomarkers and bacterial infection. Data regarding diagnostic accuracy for each biomarker in predicting bacterial cause of exacerbation were extracted and summarised. We used to QUADAS-2 tool to assess risk of bias.

**Results:**

Of 509 papers identified, 39 papers evaluating 61 biomarkers were eligible for inclusion. Moderate quality evidence was found for associations between serum C-reactive protein (CRP), serum procalcitonin (PCT), sputum interleukin (IL)-8 and sputum tumour necrosis factor alpha (TNF-α), and the presence of bacterial pathogens in the sputum of patients with AECOPD. Having bacterial pathogens was associated with a mean difference (higher) CRP and PCT of 29.44 mg/L and 0.76 ng/mL respectively. There was inconsistent or weak evidence for associations between bacterial AECOPD and higher levels of sputum IL-1β, IL-6, myeloperoxidase (MPO) and neutrophil elastase (NE). We did not find any consistent evidence of diagnostic value for other biomarkers.

**Conclusions:**

There is moderate evidence from heterogeneous studies that serum CRP and PCT are of value in differentiating bacterial from non-bacterial AECOPD, and little evidence for other biomarkers. Further high-quality research on the role of biomarkers in identifying bacterial exacerbations is needed.

**Supplementary Information:**

The online version contains supplementary material available at 10.1186/s12890-022-01958-4.

## Background

Chronic Obstructive Pulmonary Disease (COPD) is the third leading cause of death worldwide and incidence is predicted to increase each year until at least 2030 [1]. Exacerbations are defined as “acute worsening of respiratory symptoms that result in additional therapy” and carry with them significant risk of morbidity and mortality, as well as worsening disease prognosis [2]. The GOLD international guideline recommends use of antibiotics for acute exacerbations of COPD (AECOPD) in patients with increased sputum purulence who have one or both of increased dyspnoea and increased sputum volume, as well as patients that require mechanical ventilation [3]. U.K. NICE guidance advises prescribers to consider the severity of symptoms, particularly sputum colour changes and increases in volume or thickness, whether they may need to go to hospital, previous exacerbation and hospitalisation history, and the risk of developing complications, previous sputum culture results, and the risk of antimicrobial resistance when considering whether or not to prescribe antibiotics for patients with AECOPD [4]. As a result, although less than 50% of exacerbations involve bacterial infection, around three quarters of those managed in primary care, and nearly all of those managed in hospital, are treated with antibiotics [5–7]. Inappropriate use of antibiotics is the key driver of antimicrobial resistance, a major public health threat, and can also lead to adverse effects, damaging changes in the microbiome, wasted resources, and distraction from more appropriate therapy. There is therefore an urgent need to develop approaches for better diagnosing bacterial infection in a timely fashion. One such approach is the use of biomarkers.

Biomarkers are defined as “A defined characteristic that is measured as an indicator of normal biological processes, pathogenic processes or responses to an exposure or intervention” [8]. In the context of AECOPD, we are referring primarily to the measurement of molecules that are either directly from a pathogen (e.g. nucleic acid) or result from the host’s response to the pathogen, and can be measured from blood or respiratory secretions. Host responses could be either broad inflammatory markers (such as C-reactive protein) or more specific immune or inflammatory markers. New molecular technologies have improved our ability to detect and quantify biomarkers and led to a proliferation of studies on their diagnostic properties.

## Methods

### Design and aim

This systematic review and meta-analysis aims to assess and summarise and evaluate the evidence for the diagnostic value of serum and sputum biomarkers in the differentiation of bacterial versus non-bacterial acute exacerbations of COPD (AECOPD).

### Eligibility criteria

Cross-sectional, cohort and randomised controlled studies that describe associations between serum or sputum molecular or cellular biomarkers and evidence of bacterial infection in people with acute exacerbation of COPD were eligible for inclusion. We excluded animal studies, publications of abstracts only, case reports, letters, comments and reviews, and publications in languages other than English. Our review protocol was not published.

### Information sources and search strategy

We searched Embase and Medline from inception (1947 for Embase, 1946 for Medline) until 19^th^ March 2020 using a search strategy that included terms for COPD, inflammation & inflammatory markers, bacterial infection and exacerbation (Additional file 1: figure S1). The results were screened for duplications, the inclusion/exclusion criteria applied, and the remaining titles & abstracts were screened for relevancy, requesting full papers where necessary. Uncertainties were screened by a second reviewer and resolved through discussion.

### Outcomes

The main outcome was the biomarker concentration in participants defined as having a bacterial exacerbation compared with those defined as having a non-bacterial exacerbation. Where provided, we also extracted the test characteristics (sensitivity, specificity, positive predictive value, negative predictive value) at various biomarker cut points.

### Risk of bias and assessing the quality of the evidence

Risk of bias was assessed using the QUADAS-2 tool [9]. This tool uses four key domains (patient selection, index test, reference standard, flow and timing) to evaluate the risk of bias, with the first three domains also used to evaluate applicability. Signalling questions are used to aid judgements of risk of bias and applicability. The overall quality of the evidence was then assessed qualitatively, with size of cohort, selection criteria, risk of bias, definitions of COPD + exacerbation + bacterial exacerbation and data completeness all contributing to this assessment.

### Data extraction

The following data were extracted from the full-text versions of included papers: year published; country in which the study was performed; setting; how the population was defined; how COPD was defined; how exacerbation was defined; number of participants and data completeness; media of samples; biomarkers studied; definition of bacterial exacerbation; results, including average concentrations of biomarkers and suggested cut-offs.

### Data synthesis

The data were synthesised descriptively, with each biomarker being assessed individually. For biomarkers where there were sufficient data, mean biomarker concentration in patients with and without evidence of bacterial infections were compared using random-effects meta-analysis estimated using restricted maximum likelihood, with absolute mean differences calculated and the results displayed using forest plots. Stata (v16.1) software was used for the meta-analysis.

## Results

**Fig. 1:**
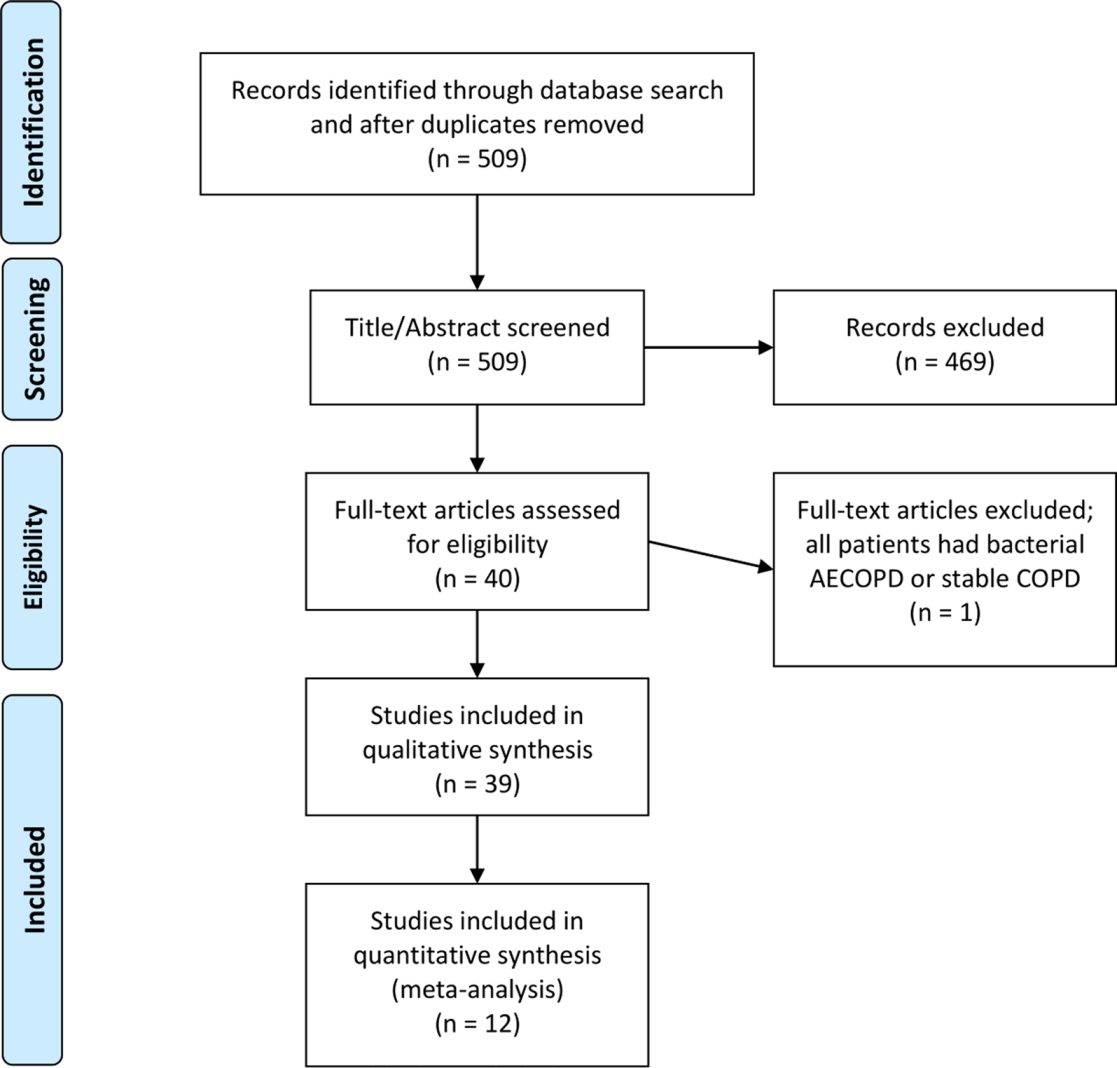
PRISMA flow diagram of literature search results

Following de-duplication and application of inclusion/exclusion criteria our search identified 509 papers. A review of the title and abstract of these 509 papers led to the exclusion of a further 469 papers. Reasons for exclusion were: the paper did not investigate patients with AECOPD (n = 108), did not assess the diagnostic accuracy of biomarkers (n = 264) or did not assess the properties of biomarkers in differentiating bacterial versus non-bacterial AECOPD (n = 97). A further one study was excluded during full text screening because it did not differentiate patients with acute exacerbations from patients with stable disease, leaving 39 studies which were included (Fig. 1).

Study publication dates ranged from 1998 to 2019. Four [10–13] were in an ICU setting, 24 [14–37] were hospital inpatient (not ICU), one [38] was both inpatient and outpatient, nine [39–47] were just outpatient, and one [48] did not specify the setting. In several studies, multiple exacerbation events were recorded for single patients, leading to a maximum of 736 [26] and minimum of 14 [47] exacerbation events reported in the studies.

Risk of bias was assessed using the QUADAS-2 tool (Table 1); for patient selection, eight [11, 16, 19, 24, 32, 37, 42, 48] and 22 [15, 17, 18, 21–23, 25–29, 31, 35, 36, 38–41, 43, 45–47] studies were assessed as being at high and unclear risk of bias respectively. For the index test, one [19] and three [27, 30, 48] studies were assessed as being at high and unclear risk of bias respectively; and reference standard, five[19, 27, 28, 37, 45] and 18 [14–17, 22, 24–26, 30, 32, 33, 35, 36, 38, 41, 43, 47, 48] studies were assessed as being at high and unclear risk of bias respectively. Only 14 (36%) [18–21, 23, 25, 27, 33, 35, 37–39, 41] studies excluded patients that had been prescribed antibiotics and corticosteroids prior to sample collection. Five (13%) studies only excluded participants who had been prescribed antibiotics [16, 17, 31, 36, 42] and another two (5%) only participants who had been prescribed corticosteroids [40, 47]. 17 (44%) [10–15, 22, 24, 26, 28–30, 32, 34, 44–46] studies did not exclude patients who had taken either antibiotics and/or systemic steroids prior to sample collection.

**Table 1:**
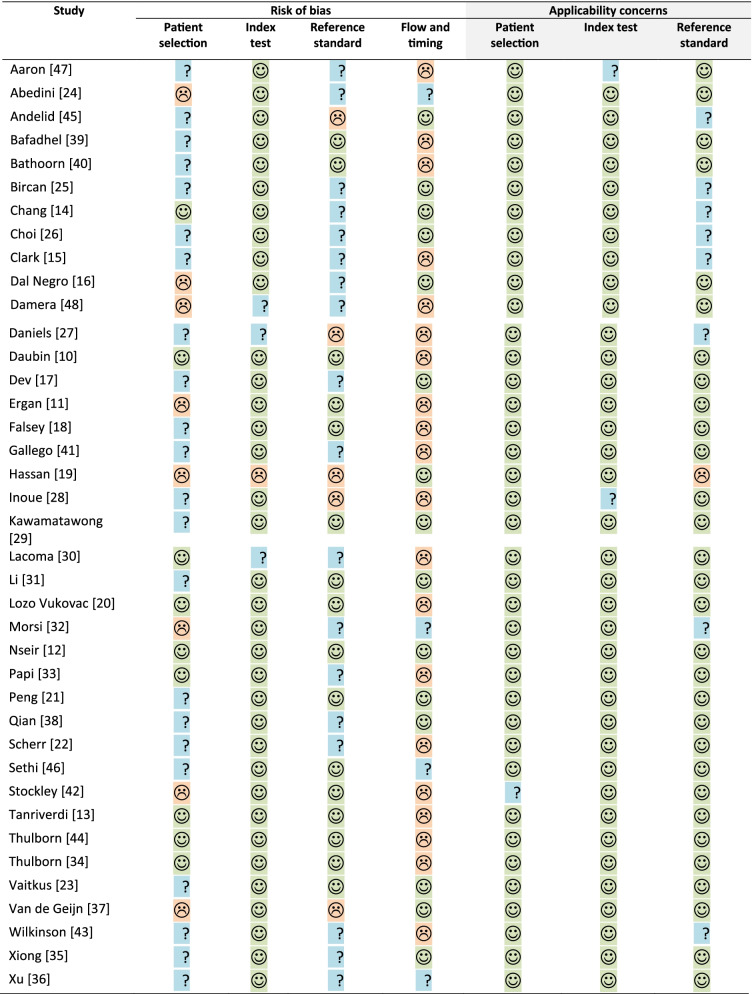
QUADAS-2 table to assess risk of bias

COPD diagnosis was based on Global Initiative for Chronic Obstructive Pulmonary Disease (GOLD) criteria in 24 (62%) studies [10, 11, 13, 14, 16, 17, 20–24, 26–29, 32–35, 37–39, 41, 45], national thoracic society guidelines in six [12, 30, 31, 36, 43, 47] studies, ‘physician diagnosis’ in three [17–19] studies, and was not described for six [15, 40, 42, 44, 46, 48] studies.

Eight (21%) studies [10, 13, 18, 26, 28, 30, 33, 39] used the GOLD definition of an exacerbation event (“acute worsening of respiratory symptoms that results in additional therapy”), and 26 (67%) studies [11, 12, 14–16, 19–25, 27, 31, 32, 34–38, 40, 42–46] described exacerbations as ‘worsening of respiratory symptoms’, with no mention of need for additional therapy. Five [17, 29, 41, 47, 48] studies did not indicate how they defined exacerbation events.

Most studies used growth of pathogenic bacteria from respiratory specimens to define bacterial exacerbations, but many of these did not provide detailed descriptions. Three [22, 46, 47] studies defined bacterial growth as growth of a ‘new strain’, six [10, 15, 19, 25, 28, 37] studies used a combination of clinical observations, sputum culture, X-ray images and lab results; and two [43, 48] studies did not state how they defined bacterial exacerbation.

The 39 included studies evaluated 61 biomarkers (27 biomarkers that were evaluated in both serum and sputum samples, an additional 28 that were only evaluated in serum and an additional 6 that were only evaluated in sputum) giving a total of 55 serum and 33 sputum biomarkers (Table 2). For most biomarkers there was insufficient data to draw meaningful conclusions. Three serum biomarkers (CRP, PCT and white blood cell count) and six sputum biomarkers (IL-8, TNF-α, IL-1β, IL-6, MPO and NE) had data from more than two studies and the findings relating to these biomarkers are summarised below.

**Table 2:** Sputum and serum biomarkers identified

Serum	Sputum
Albumin, Amino acids, B lymphocyte %, Basophil %, CCL2, CCL3, CCL4, CCL13, CCL17, CD16-neg T lymphocytes %, CD4-pos T lymphocyte %, CD64, CD64 index of granulocytes, CD64% expression, Copeptin, CRP, CXCL10, CXCL11, ECP, Eosinophil count, Eosinophil %, Glucose, GMCSF, IFN-γ, IL-10, IL-13, IL-1β, IL-5, IL-6, IL-8, Immature granulocytes %, LDH, Leukocyte count, Lymphocyte count, Lymphocyte %, MPO, Neopterin, Neutrophil count, Neutrophil %, NE, N/L ratio, PSP/reg, Plasma cell %, PCT, ROS, SAA, sICAM, sTREM-1, SPD, T and NK lymphocytes %, T-lymphocyte %, TNF-α, TNFRI, TNFRII, WBC count	Albumin, CCL13, CCL17, CCL3, CCL4, CCL5, CCL2, CRP, CXCL10, CXCL11, ECP, Eosinophil count, Glucose, IL-1B, IL-5, IL-6, IL-6R, IL-8, LDH, LTB4, Lymphocyte count, MCP-1, MPO, Neopterin, Neutrophil %, Neutrophil count, NE, PTX3, pH, ROS, TNF-a, TNFRI, TNFRII

*CCL* chemokine ligand, *CD* cluster of differentiation, *CRP* C-reactive protein, *CXCL* C-X-C motif chemokine, *ECP* eosinophil cationic protein, *GMCSF* granulocyte–macrophage colony-stimulating factor, *IFN* interferon, *IL* interleukin, *LDH* lactate dehydrogenase, *LTB4* leukotriene-B4, *MCP-1* monocyte chemoattractant protein-1, *MPO* myeloperoxidase, *NE* neutrophil elastase, *N/L* neutrophil/lymphocyte, *PSP/reg* pancreatic stone protein/regenerating protein, *PCT* procalcitonin, *PTX3* pentraxin 3, *ROS* reactive oxygen species, *SAA* serum amyloid A, *sICAM* soluble intercellular adhesion molecule, *sTREM-1* soluble myeloid cell trigger receptor-1, *SPD* surfactant protein D, *TNF* tumour necrosis factor, *TNFR* tumour necrosis factor receptor, *WBC* white blood cell

### Serum markers

#### Serum C-reactive protein (CRP)

We identified 28 studies that included an assessment of serum C-reactive protein (CRP) to determine bacterial aetiology in AECOPD (Table 3). Most studies were small, with 17 having fewer than 85 exacerbation events. 18 studies provided quantitative data, of which 15 (83%) reported higher levels of serum CRP in bacterial versus non-bacterial exacerbations, and the difference was statistically significant in 12 [11, 13, 20, 23, 27, 31, 35–37, 39–41]. Of the 10 papers that did not provide accessible numerical data, two reported a significant association between CRP level and bacterial AECOPD [19, 21], and another two reported a significant association between CRP level and purulent sputum, a proxy for bacterial AECOPD [25, 42]. A further three [14, 38, 45] did not find any significant association between CRP and bacterial AECOPD. One paper found that isolation of new pathogenic strains in sputum was associated with a greater increase in CRP compared to pre-exacerbation levels than for non-bacterial AECOPD, pre-existing strain isolation or other strain isolation [46].

**Table 3:** Description of studies assessing the properties of C-Reactive Protein (CRP) for identifying bacterial vs non-bacterial AECOPD

Author (reference)	Setting	Antibiotics or Steroids in the exclusion criteria (length of exclusion)	GOLD Stage of Cohort	Definition of bacterial cause of exacerbation	Number of exacerbations	Number of exacerbations with bacterial cause	Mean CRP (SD; mg/L) Bacterial exacerbation	Mean CRP (SD; mg/L) Non-bacterial exacerbation
Abedini [24]	H	Neither	I–IV	Positive sputum culture	68	26	–	–
Andelid [45]	OP	Neither	I–IV	Positive sputum culture	29	14	–	–
Bafadhel [39]	OP	Antibiotics & Steroids	I–IV	Positive sputum culture OR total aerobic count > 10^7^ CFU	158	84	13 (41)*	5 (15)*
Bathoorn [40]	OP	Steroids	–	High semi-quantitative growth density of PPM on sputum culture	37	8	9.08 (4.56–26.2)*	2.6 (1.4–15.3)*
Bircan [25]	H	Antibiotics (1 week) & Steroids (2 weeks)	-	Purulent sputum	51	–	–	–
Chang [14]	H	Neither	I–IV	Positive sputum culture	72	30	–	–
Clark [15]	H	Neither	–	Positive sputum culture	195	66	20 (3–39)*	8 (3–28)*
Daniels [27]	H	Antibiotics & Steroids	I–IV	Positive sputum culture	243	142	33.0 (9.75–88.25)*	17.0 (5–61.0)*
Dev [17]	H	Antibiotics	–	Positive sputum culture	50	29	103 (98)	92 (90)
Ergan [11]	ICU	Neither	III–IV	Sputum sample > 10^6^ CFU OR endotracheal/tracheal aspirate > 10^5^ CFU OR mini-bronchoalveolar lavage > 10^4^ CFU	52	16	73 (40–87)*	53 (21–104)*
Gallego [41]	OP	Antibiotics & Steroids	I–IV	Positive sputum culture	265	167	58.3 (21.0–128.2)*	Viral = 37.3 (18.6–79.1)* Non-pathogenic = 36.4 (10.8–93.7)*
Hassan [19]	H	Antibiotics (2 weeks) & Steroids (2 weeks)	I–IV	Combination of clinical observations, sputum culture, X-ray images & lab results	30	22	22.27 (0.968–1)^+^	–
Kawamatawong [29]	H	Neither	II–IV	Positive sputum culture for aerobic organisms	62	29	41.62 (4.16–274.8)*	Viral = 37.46 (23.69–700.0)* Non-pathogenic = 15.56 (1.0–238.5)*
Lacoma [30]	H	Neither	–	Positive sputum culture	161	76	88.61 (34.89–201.12)*	88.66 (38.04–164.35)*
Li [31]	H	Antibiotics (4 weeks)	–	Positive sputum culture	164	98	73.81 (18.27)	7.91 (3.01)
Lozo Vukovac [20]	H	Antibiotics (2 months) & Steroids (2 months)	II–IV	Bronchoalveolar aspirate > 10^3^ CFU/mL	84	60	29.4 (11.2)	16.8 (11.6)
Morsi [32]	H	Neither	–	Positive sputum culture	31	19	–	–
Nseir [12]	ICU	Neither	II–IV	Endotracheal aspirate > 10^6^ CFU/mL	98	40	56 (92)	56 (65)
Peng [21]	H	Antibiotics & Steroids	-	≥ 1 PPMs in excess (≥ 1 log) of normal microbiological flora in sputum OR PPMs reaching a level of absolute growth > 10^6^ CFU/mL (*Strep. pneumoniae* > 10^5^ CFU/mL sufficient)	81	55	–	–
Qian [38]	H & OP	Antibiotics & Steroids	-	Positive sputum culture	150	82	–	–
Scherr [22]	H	Neither	I–IV	Positive sputum culture	108	65	45.7 (52.6)	46.4 (63.2)
Sethi [46]	OP	–	I–IV	Positive sputum culture	150	84	–	–
Stockley [42]	OP	Antibiotics (4 weeks)	–	Positive sputum culture	121	86	–	–
Tanriverdi [13]	ICU	Neither	–	Positive sputum culture > 10^5^ CFU/mL	77	28	106.7 (83.7)	105.6 (101.5)
Vaitkus [23]	H	Antibiotics (1 month) & Steroids (1 month)	–	Positive sputum culture > 10^6^ CFU/mL	40	18	23.5 (20.8)	5.8 (2.5)
Van de Geijn [37]	H	Antibiotics (2 weeks) & Steroids (2 weeks)	I–IV	Combination of clinical observations, sputum culture, X-ray images & lab results	45	22	114.95 (118.93)	21.17 (27.20)
Xiong [35]	H	Antibiotics (2 weeks) & Steroids (2 weeks)	I–IV	Positive sputum culture	78	38	56.65 (31.65)	19.62 (8.78)
Xu [36]	H	Antibiotics (4 weeks)	–	Positive sputum culture > 10^7^ CFU/mL	60	26	58.87 (9.77)	18.66 (3.98)

28 studies were identified to evaluate the relationship between CRP and AECOPD aetiology

*GOLD* global initiative for chronic obstructive lung disease, *CRP* C-reactive protein, *OP* outpatient, *H* hospital, *ICU* intensive care unit, *CFU* colony forming units, *PPM* potentially pathogenic microorganisms, *BA-aspirate* Bronchoalveolar aspirate. *, Median (IQR); -, information not provided

**Fig. 2:**
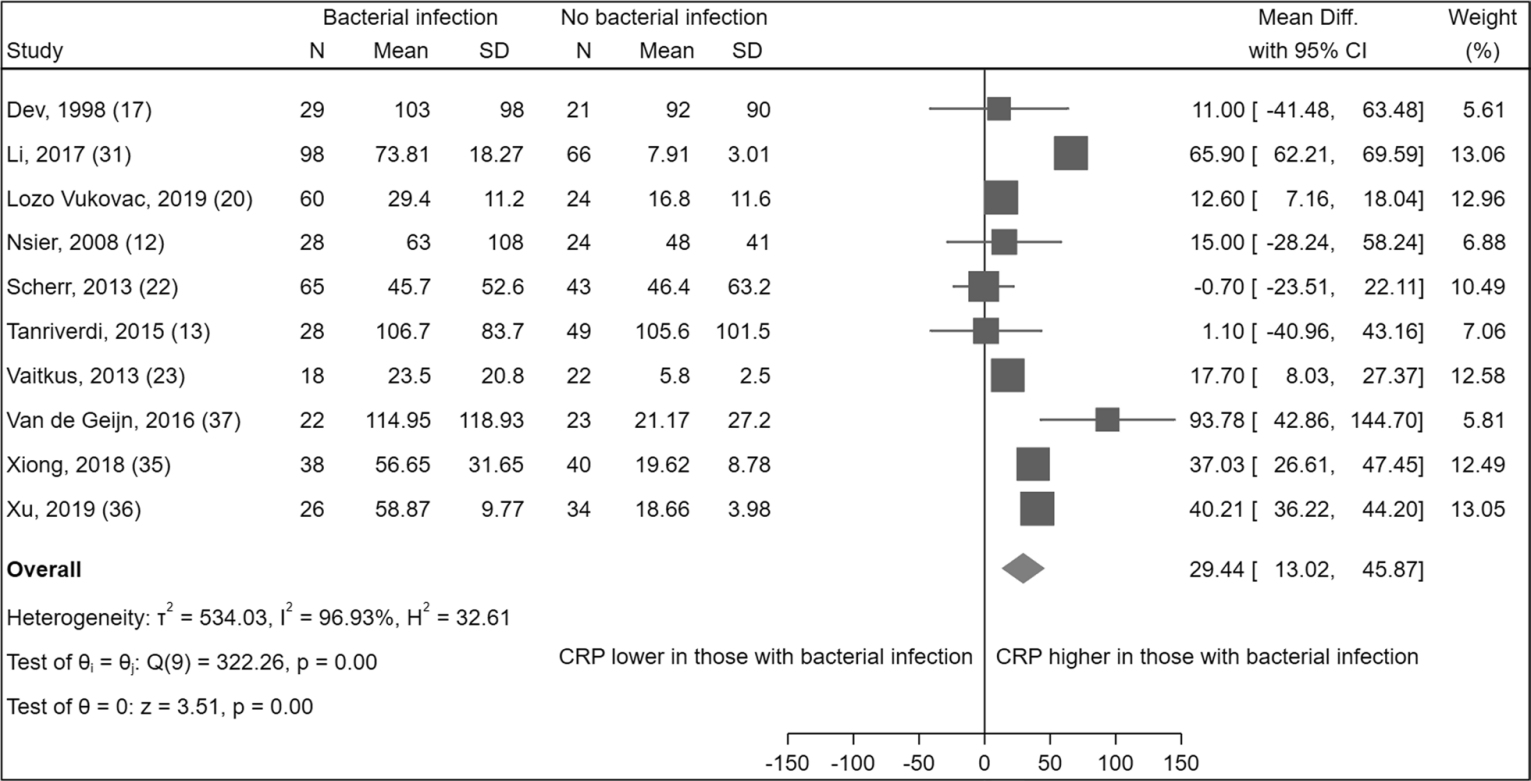
Forest plot of the difference in mean CRP values in AECOPD patients with and without a bacterial infection

Only 10 [12, 13, 17, 20, 22, 23, 31, 35–37] studies provided a mean and standard deviation CRP for each group, and therefore could be included in a meta-analysis. The meta-analysis found that bacterial exacerbations were associated with significantly higher CRP values, with a weighted mean difference of 29.44 mg/L (Fig. 2). However, high heterogeneity with I^2^ = 96.93% was observed. Subgroup analysis by setting, use of antibiotics or steroids, or definitions of COPD, exacerbation or bacterial infection did not reduce heterogeneity.

Several authors have suggested cut-points for use in clinical practice to best identify bacterial vs non-bacterial AECOPD (Table 4). The cut-points range from 5 to 110 mg/L and tend to vary by setting. The best overall test characteristics were reported by Hassan et al. [19] who found that a cut point of 15 mg/L resulted in a sensitivity and specificity of 95.5% and 100% respectively for identifying bacterial pathogens in a study of 30 patients with AECOPD in a hospital inpatient setting.

**Table 4:** Suggested C-reactive protein (CRP) cut-off values to predict bacterial versus non-bacterial AECOPD

Author	Setting	Number of Exacerbations	Number of Exacerbations with Bacterial Cause	Cut-off / mg/L	Sensitivity / %	Specificity / %	Positive Predictive Value / %	Negative Predictive Value / %
Bafadhel [39]	OP	158	84	10	60	70	–	–
Bircan [25]	H	51	–	10	72.5	100	–	–
Hassan [19]	H	30	22	15	95.5	100	–	–
Peng [21]	H	81	55	19.65	78	85	–	–
Peng (with mucoid sputum)				15.21	81	77	–	–
Sethi [46]	OP	150	84	2.37	61.8	68.3	39.6	84.1
Tanriverdi [13]	ICU	77	28	1.5	54	52	–	–
				42	82	35	–	–
				110	35	80	–	–
Van de Geijn [37]	H	45	22	5	91	39	–	–
Xiong [35]	H	78	38	31.68	68.42	85.00	89.81	58.35

–, information not provided

#### Serum procalcitonin (PCT)

17 papers described the association between serum Procalcitonin (PCT) and evidence of a bacterial infection in AECOPD (Table 5). Of the 15 that provided numerical data, 11 (65%) found higher PCT concentrations in patients with bacterial AECOPD compared to non-bacterial AECOPD, and six of these reported a statistically significant difference [13, 31, 35–38]. Of the two papers that did not include numerical data, one reported that PCT concentrations > 0.5 ng/mL and “positive CRP” (which was not defined) were both associated with positive sputum culture, and that PCT < 0.5 ng/mL was strongly associated with non-bacterial AECOPD [24], whilst the other reported no significant difference in PCT concentrations between bacterial and non-bacterial AECOPD [14].

**Table 5:** Description of studies assessing the properties of Procalcitonin (PCT) for identifying bacterial vs non-bacterial AECOPD

Author	Setting	Antibiotics or Steroids in the exclusion criteria (length of exclusion)	GOLD Stage of Cohort	Definition of Bacterial Cause of Exacerbation	Number of Exacerbations	Number of Exacerbations with Bacterial Cause	Mean PCT (SD; ng/mL) Bacterial Exacerbation	Mean PCT (SD; ng/mL) Non-bacterial Exacerbation
Abedini [24]	H	Neither	I–IV	Positive sputum culture	68	26	+	+
Bafadhel [39]	OP	Antibiotics & Steroids	I–IV	Positive sputum culture OR total aerobic count > 10^7^ CFU	158	84	0.06 (0.04)*	0.06 (0.04)*
Chang [14]	H	Neither	I–IV	Positive sputum culture	72	30	+	+
Daniels [27]	H	Antibiotics & Steroids	I–IV	Positive sputum culture	243	142	0.06 (0.04–0.11)*	0.06 (0.04–0.08)*
Daubin [10]	ICU	Neither	I–IV	Positive Gram stain OR tracheobronchial aspirate > 10^5^ CFU/mL OR positive blood culture without extrapulmonary focus	39	5	0.081 (0.062–0.189)*	0.098 (0.065–0.170)*
Ergan [11]	ICU	Neither	III–IV	Sputum sample > 10^6^ CFU OR endotracheal/tracheal aspirate > 10^5^ CFU OR mini-bronchoalveolar lavage > 10^4^ CFU	52	16	0.41 (0.12–0.99)*	0.18 (0.07–0.37)*
Falsey [18]	H	Antibiotics & Steroids	+	Positive serum & sputum cultures, nose & throat swab & urine analysis	104	32	0.32 (0.57)	0.20 (0.66)
Kawamatawong [29]	H	Neither	II–IV	Positive sputum culture for aerobic pathogens	62	29	0.30 (0.04–17.6)*	Viral = 0.026 (0.07–18.48)* Non-pathogenic = 0.09 (0.03–19.29)*
Lacoma [30]	H	Neither	+	Positive sputum culture	161	76	0.10 (0.07–0.22)*	0.10 (0.06–0.21)*
Li [31]	H	Antibiotics (4 weeks)	+	Positive sputum culture	164	98	2.52 (2.89)	0.17 (0.07)
Nseir [12]	ICU	Neither	II–IV	Endotracheal aspirate > 10^6^ CFU/mL	98	40	0.67 (1.3)	0.66 (1.2)
Qian [38]	H & OP	Antibiotics & Steroids	+	Positive sputum culture	150	82	0.26 (0.12)	0.17 (10.08)
Scherr [22]	H	Neither	I–IV	Positive sputum culture	108	65	0.29 (0.6)	0.14 (0.12)
Tanriverdi [13]	ICU	Neither	+	Positive sputum culture > 10^5^ CFU/mL	77	28	2.93 (6.6)	0.75 (1.29)
Van de Geijn [37]	H	Antibiotics (2 weeks) & Steroids (2 weeks)	I–IV	Combination of clinical observations, sputum culture, X-ray images & lab results	45	22	1.15 (2.88)	0.05 (0.03)
Xiong [35]	H	Antibiotics (2 weeks) & Steroids (2 weeks)	I–IV	Positive sputum culture	78	38	1.63 (0.85)	0.35 (0.27)
Xu [36]	H	Antibiotics (4 weeks)	+	Positive sputum culture	60	26	0.35 (0.03)	0.14 (0.02)

17 studies were identified to evaluate the relationship between PCT and AECOPD aetiology

*OP* outpatient, *H* hospital, *ICU* intensive care unit, *CFU* colony forming units. *, Median (IQR); + , information not provided

**Fig. 3:**
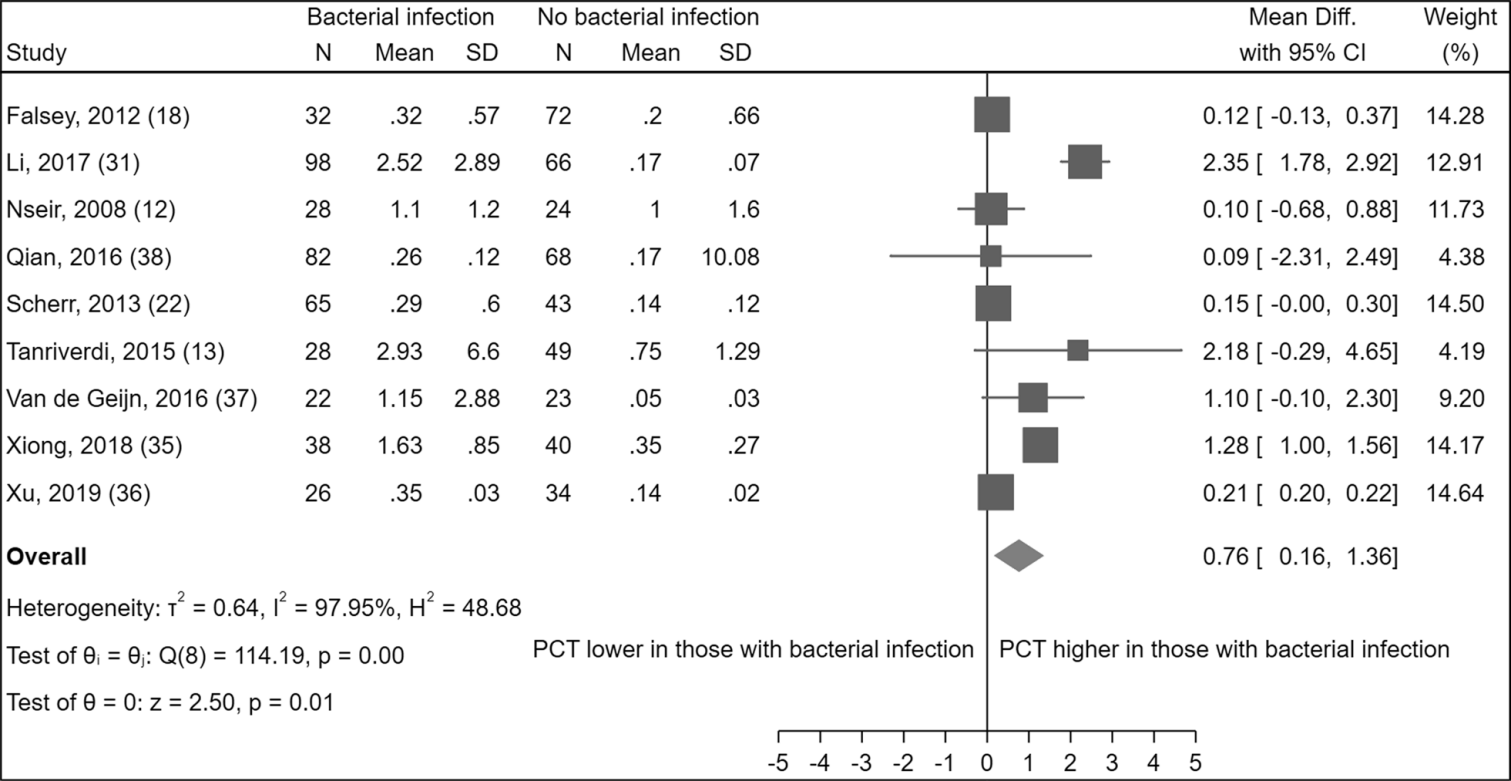
Forest plot of the difference in mean PCT values in AECOPD patients with and without a bacterial infection

We were able to extract mean and standard deviation PCT levels for those with and without evidence of a bacterial infection from nine [12, 13, 18, 22, 31, 35–38] studies. Combining these data using meta-analysis we found higher mean PCT in those with a bacterial exacerbation, with a weighted mean difference of 0.76 ng/mL (95% CI: 0.16, 1.36 ng/mL; Fig. 3). High heterogeneity (I^2^ = 97.95%) was also observed. Again, subgroup analysis by setting, use of antibiotics or steroids, or definitions of COPD, exacerbation or bacterial infection did not reduce heterogeneity.

The authors of these papers describe cut-points ranging from 0.03–1.03 ng/mL to identify bacterial vs non-bacterial AECOPD (Table 6). The study with the greatest area under receiver operating characteristic curve (ROC) used a cut-point of 0.76 ng/mL resulting in a specificity of 92.5% and sensitivity of 78.95%, with area under the curve in diagnosing bacterial AECOPD of 0.941 [35].

**Table 6:** Suggested procalcitonin (PCT) cut-offs to predict bacterial versus non-bacterial AECOPD

Author	Setting	Number of Exacerbations	Number of Exacerbations with Bacterial Cause	Cut-off / ng/mL	Sensitivity / %	Specificity / %	Positive Predictive Value / %	Negative Predictive Value / %
Ergan [11]	ICU	52	16	0.25	63	67	0.45	0.80
Nseir (all patients) [12]	ICU	98	40	0.5	45	70.7	51	65
Nseir (Abx excluded)				0.5	53.5	87.5	83.3	61.7
Tanriverdi [13]	ICU	77	28	0.40	61	67	–	–
				1.03	43	83	–	–
				0.10	75	40	–	–
Van de Geijn [37]	H	45	22	0.03	91	36	–	–
Xiong [35]	H	78	38	0.76	78.95	92.50	96.74	74.21

*Abx* antibiotics; -, information not provided

#### Serum WBC

Five studies, ranging in size from 60 to 150 participants and 26 to 82 bacterial exacerbations, examined associations between serum WBC count and bacterial (versus non-bacterial) AECOPD [14, 29, 35, 36, 38]. Three were in hospital inpatient settings and two were in emergency departments. None of the studies demonstrated a statistically significant association.

### Sputum markers

#### Sputum IL-8

Seven studies compared sputum IL-8 levels in bacterial and non-bacterial AECOPD. The studies varied in size from 14 [47] to 84 [46] participants, with between 14 and 158 exacerbation events. Four [16, 23, 39, 40] of these studies found significantly higher IL-8 levels associated with bacterial AECOPD (Table 7). One study [43] reported a positive association between the rise in airway bacterial load and the rise in sputum IL-8 concentrations during exacerbation, although this is not the same as differentiating bacterial from non-bacterial exacerbations. Another study [46] found no significant difference in sputum IL-8 concentrations between those with a new strain, and those with a pre-existing strain, other strain or non-bacterial AECOPD.

**Table 7:** Description of studies assessing the properties of Sputum IL-8 for identifying bacterial vs non-bacterial AECOPD

Author	Setting	Antibiotics or Steroids in the exclusion criteria (length of exclusion)	GOLD Stage of Cohort	Definition of Bacterial Cause of Exacerbation	Number of Exacerbations	Number of Exacerbations with Bacterial Cause	Mean IL-8 (SD) Bacterial Exacerbation	Mean IL-8 (SD) Non-bacterial Exacerbation
Aaron [47]	OP	Steroids	–	Positive sputum culture of pathogen not cultured at baseline	14	1	–	–
Bafadhel [39]	OP	Antibiotics & Steroids	I–IV	Positive sputum culture OR total aerobic count > 10^7^ CFU	158	84	8926 pg/mL	3221 pg/mL
Bathoorn [40]	OP	Steroids	–	High semi-quantitative growth density of PPM on sputum culture	37	8	7.78 μg/mL	1.74 μg/mL
Dal Negro [16]	H	Antibiotics	II	Positive sputum culture > 10^6^ CFU/mL	124	CB = 28 PA = 20	CB = 16,599.6 pg/mL PA = 16,087.4 pg/mL	NI = 8201.8 pg/mL V = 9996.0 pg/mL
Sethi [46]	OP	–	I–IV	Molecular typing of strains from sputum	150	84	–	–
Vaitkus [23]	H	Antibiotics (1 month) & Steroids (1 month)	–	Positive sputum culture > 10^6^ CFU/mL	40	18	468 pg/mL	410 pg/mL
Wilkinson [43]	OP	Antibiotics	–	–	39	–	–	–

**7** studies were identified to evaluate the relationship between sputum IL-8 and AECOPD aetiology

OP, Outpatient; H, Hospital; CFU, Colony forming units; PPM, Potentially pathogenic microorganisms; CB, common bacteria; PA, *Pseudomonas aeruginosa*; NI, Not infective; V, Viral. -, information not provided

Importantly, there was large variation in the detected levels of sputum IL-8 between studies. One [40] reported a mean IL-8 of 7.78 μg/mL in bacterial exacerbations, while another [23] reported a mean concentration of 468 pg/mL in those with bacterial exacerbations. Five [16, 23, 39, 43, 46] studies had mean sputum IL-8 concentrations between 0.468 and 16.6 ng/mL, whilst one [47] had mean concentration of 100 ng/mL, the other [40] 7.78 μg/mL. The variation in concentrations did not appear to be explained by setting or testing procedure, as there was no important difference between methods of sputum induction nor the concentration of Il-8 in studies that used ELISA (n = 3) and studies that did not (n = 4).

#### Sputum TNF-α

The association between sputum TNF-α concentrations and evidence of bacterial exacerbation was studied in five papers (Table 8). The studies were performed in an outpatient setting [39, 40, 46, 47], or only included patients with stage II AECOPD according to GOLD classification [16], and so included patients with mild-moderate exacerbations.

**Table 8:** Description of studies assessing the properties of Tumour Necrosis Factor-alpha [TNF-α) for identifying bacterial vs non-bacterial AECOPD

Author	Setting	Antibiotics or Steroids in the exclusion criteria (length of exclusion)	GOLD Stage of Cohort	Definition of Bacterial Cause of Exacerbation	Number of Exacerbations	Number of Exacerbations with Bacterial Cause	Mean TNF-α (SD; pg/mL) Bacterial Exacerbation	Mean TNF-α (SD; pg/mL) Non-bacterial Exacerbation
Aaron [47]	OP	Steroids	–	Positive sputum culture of pathogen not cultured at baseline	14	1	–	–
Bafadhel [39]	OP	Antibiotics & Steroids	I–IV	Positive sputum culture OR total aerobic count > 10^7^ CFU	158	84	89.7 [61.9 to 129.9]	7.5 [5.2 to 10.8]
Bathoorn [40]	OP	Steroids	–	High semi-quantitative growth density of PPM on sputum culture	37	8	56.8 (43.3–69.7)*	3.43 (1.60–7.73)*
Dal Negro [16]	H	Antibiotics	II	Positive sputum culture > 10^6^ CFU/mL	124	CB = 28 PA = 20	CB = 721.6 (1186.0)^$^ PA = 2417.6 (1485.3)^$^	NI = 112.1 (119.5)^$^ V = 181.8 (125.6)^$^
Sethi [46]	OP	–	I–IV	Positive sputum culture	150	84	–	–

5 studies were identified to evaluate the relationship between TNF-α and AECOPD aetiology

*OP* outpatient, *H* hospital, *CFU* colony forming units, *PPM* potentially pathogenic microorganisms. *, Median (IQR); -, information not provided; ^$^, mean (IQR); [x] where x = 95% confidence intervals

Average sputum TNF-α was significantly higher in bacterial exacerbations than non-bacterial in four papers [16, 39, 40, 46]. The other study only identified one patient with bacterial AECOPD and therefore could not draw meaningful conclusions [47]. One of these studies further classified causes of exacerbation into common bacteria, *Pseudomonas aeruginosa*, viral and non-infective. They found mean TNF-α to be significantly higher in patients with *Pseudomonas aeruginosa* infections than the other three groups [16].

#### Sputum IL-1B

Three studies investigated the utility of sputum IL-1β as a marker for bacterial AECOPD [16, 39, 48]. One study found that sputum IL-1β had an area under ROC of 0.89 for detecting bacterial exacerbations, and that a cut-point of 125 pg/mL had sensitivity and specificity of 90% and 80% respectively [39]. Another found a significant association between ≥ twofold increase (compared to stable state) in sputum IL-1β concentrations (IL-1β^+^ event) and bacterial exacerbations [48]. The third reported an area under receiver operating curve of 0.87 from use of a combination of sputum IL-8 and IL-1β for determining common bacterial from viral and non-infectious AECOPD, but only after first excluding *P. aeruginosa* infections [16].

#### Sputum IL-6

Sputum IL-6 was investigated in three studies. One study involving 45 exacerbations reported a difference in IL-6 concentrations that was not statistically significant (680 pg/mL vs. 325 pg/mL; *p* > 0.05), but a difference in percentage change that did reach statistical significance (116% vs. −16%; *p* < 0.05) [40]. Another study of 182 exacerbation events from 96 patients found that the area under ROC was 0.7 in determining bacterial from non-bacterial AECOPD [39]. In contrast, Wilkinson found that there was no significant change between stable state and AECOPD, and that there was no association between sputum IL-6 concentrations and airway bacterial load [43].

#### Sputum MPO

Three small studies investigated sputum MPO as a marker of bacterial AECOPD. Two of these were too small to draw any meaningful conclusions (29 exacerbation events [45] and 14 exacerbation events [47]). The third found significantly higher MPO concentrations in patients with bacterial versus non-bacterial AECOPD (57.7 vs. 12.6 μg/mL; *p* < 0.05) in a study involving 45 exacerbation events [40].

#### Sputum NE

Sputum NE was investigated in three studies. One study of 30 exacerbations with 13 of bacterial aetiology found an association between sputum NE concentrations and bacterial AECOPD (log difference 3.873; *p* = 0.011). In addition, NE was positively correlated with CFU load (r = 0.506; *p* = 0.005) [34]. Neither of the other two (N = 150 and 29) studies found significant associations with bacterial AECOPD, but one reported a significant association with detecting a new bacterial strain, not present in stable state, at AECOPD (new strain; *p* < 0.001) [45, 46].

## Discussion

This systematic review appraised 39 studies evaluating 61 biomarkers. Four biomarkers (serum CRP and PCT, and sputum IL-8 and TNF-α) show potential for use in differentiating bacterial from non-bacterial AECOPD. The strongest evidence was found for serum CRP, where studies performed on non-ICU inpatient populations largely suggested greater CRP concentrations in patients presenting with bacterial AECOPD. High heterogeneity in meta-analysis did not allow for reliable quantitative synthesis for serum CRP or PCT, and meta-analysis was not possible for IL-8 or TNF-α due to the low number of studies. The available evidence suggests that serum WBC count is not useful as a marker of bacterial AECOPD. The evidence for sputum IL-1β, IL-6, MPO and NE as biomarkers for detecting bacterial AECOPD is inconclusive, and there was insufficient evidence available for other biomarkers evaluated.

### Strengths and limitations

Strengths of this review include the broad aims and inclusion criteria, the systematic approach to searching and selection of studies, and the use of meta-analysis to pool data. We were able to identify 39 relevant studies from 1105 titles identified by our search strategy. However, most studies had small sample sizes with fewer than 50 bacterial exacerbation events, and only a quarter of the studies had more than 100 exacerbation events of any aetiology in their analysis. Furthermore, some of the studies did not present information in a way that enabled us to perform meta-analysis, which further limited our capabilities in drawing reliable conclusions. The quality of the evidence was mixed, with a large number of studies having a high or unclear risk of bias in terms of patient selection, but most studies being at low risk of bias in terms of the index test used. A large proportion of studies included patients that were taking antibiotics prior to giving samples, which could have reduced positive culture rates and so underestimated the number of bacterial exacerbations and therefore artificially reduced the ability of the biomarkers to correctly predict a bacterial cause based on culture positivity. In addition to this, some patients were already receiving corticosteroid treatment prior to giving samples which could reduce the concentrations of inflammatory biomarkers (such as CRP) and therefore affect the ability of these biomarkers to positively identify a bacterial cause. Furthermore, two studies did not describe how they defined ‘bacterial exacerbation’ and two studies examining the utility of CRP [19, 37] used CRP > 50 mg/L as part of their definition of bacterial AECOPD, leading to classification bias. Further limitations include the fact that initial screening was only done by one reviewer (with uncertainties discussed with a second reviewer), only two databases were searched, and that we excluded studies not published in English.

A major challenge in synthesising and interpreting these studies is the variation in setting and severity of participants. Most studies involving out-patients, and some inpatient studies, found CRP levels < 40 mg/L in those with no evidence of bacterial infection. However, six studies reported mean or median CRP levels greater than 40 mg/L (46.4 mg/L–105.6 mg/L) in culture negative patients, and correspondingly higher CRP concentrations in culture positive patients (45.7 mg/L–106.7 mg/L). The patients in these cohorts often had more severe COPD and exacerbations with worse respiratory symptoms than many of the other studies. In addition, three studies were performed on patients admitted to ICU, of which mean CRP of bacterial AECOPD was 56–106.7 mg/L, and 53–105.6 mg/L in non-bacterial AECOPD [11–13]. This variation in underlying severity likely contributed to the high level of heterogeneity found in the meta-analysis, however sub-group analyses by setting did not sufficiently reduce heterogeneity.

Mean PCT concentrations in non-bacterial AECOPD were > 0.2 ng/mL in 3 studies, although two of the studies were performed in ICU, where in one 60% of their cohort had GOLD stage III/IV disease [13] and in the other 75% of the cohort with non-bacterial AECOPD had GOLD stage IV disease [12]. Mean PCT was still reported higher in bacterial versus non-bacterial AECOPD in two of the three studies despite relatively high PCT concentrations in the non-bacterial AECOPD groups [13, 35].

Variation in the definitions used for COPD, exacerbations, and bacterial exacerbations is likely to have introduced further heterogeneity. Most of the studies used different definitions which made drawing comparisons between them difficult, and any analysis across them is limited. Despite international classification schemes being developed such as GOLD and Anthonisen [49] criteria, many studies decided to use other parameters to measure COPD & exacerbation severity. Moreover, there was large variation in the way that bacterial exacerbation was defined. The most common approach was to use a certain level of growth (CFU/mL) of a known pathogen from sputum culture, but this approach can be biased by sampling, transport and culturing techniques, and fails to differentiate between colonisation and acute infection. Indeed, almost all approaches failed to differentiate airway colonisation from acute infection. This is significant as the presence of bacteria does not indicate disease, but rather the combination of dysbiosis with key pathogens, such as non-typeable *Haemophilus influenzae*, that leads to inflammation and acute exacerbation events. Furthermore, many patients experience co-infection with more than one organism, including co-infection with bacterial and viral pathogens. In these patients it is difficult to determine the relative effects of the different pathogens, and therefore the relative need for antibiotic treatment.

## Conclusions

In conclusion, there are several biomarkers that show promise for use in differentiating bacterial from non-bacterial AECOPD, with serum CRP having the most convincing evidence. However, given the low number of studies identified and large amount of heterogeneity between studies, it is not possible to draw firm conclusions about the value of using biomarkers to identify bacterial exacerbations of COPD. Further well-powered high-quality studies are needed, especially on markers with some evidence of value such as serum CRP and PCT, and sputum IL-8, TNF-α, IL-1β, IL-6, MPO and NE.

## Supplementary Information

**Additional file 1**. **Supplementary Figure S1.** Search strategy.

## Data Availability

All data generated or analysed during this study are included in this published article.

## Abbreviations

COP: Chronic obstructive pulmonary disease
CCL: Chemokine ligand
CD: Cluster of differentiation
CRP: C-reactive protein
CXCL: C-X-C motif chemokine
ECP: Eosinophil cationic protein
GMCSF: Granulocyte–macrophage colony-stimulating factor
IFN: Interferon
IL: Interleukin
LDH: Lactate dehydrogenase
LTB4: Leukotriene-B4
MCP-1: Monocyte chemoattractant protein-1
MPO: Myeloperoxidase
NE: Neutrophil elastase
N/L: Neutrophil/lymphocyte
PSP/reg: Pancreatic stone protein/regenerating protein
PCT: Procalcitonin
PTX3: Pentraxin 3
ROS: Reactive oxygen species
SAA: Serum amyloid A
sICAM: Soluble intercellular adhesion molecule
sTREM-1: Soluble myeloid cell trigger receptor-1
SPD: Surfactant protein D
TNF: Tumour necrosis factor
TNFR: Tumour necrosis factor receptor
WBC: White blood cell
