# Supplementary figures and images for: Biomarkers to guide the use of antibiotics for acute exacerbations of COPD (AECOPD): a systematic review and meta-analysis
Source: BMC Pulm Med. 2022 May 13;22:194. doi: 10.1186/s12890-022-01958-4 (PMC9101830; doi:10.1186/s12890-022-01958-4)

**Appendix**

**Supplementary Figure S1 – Search strategy**

**
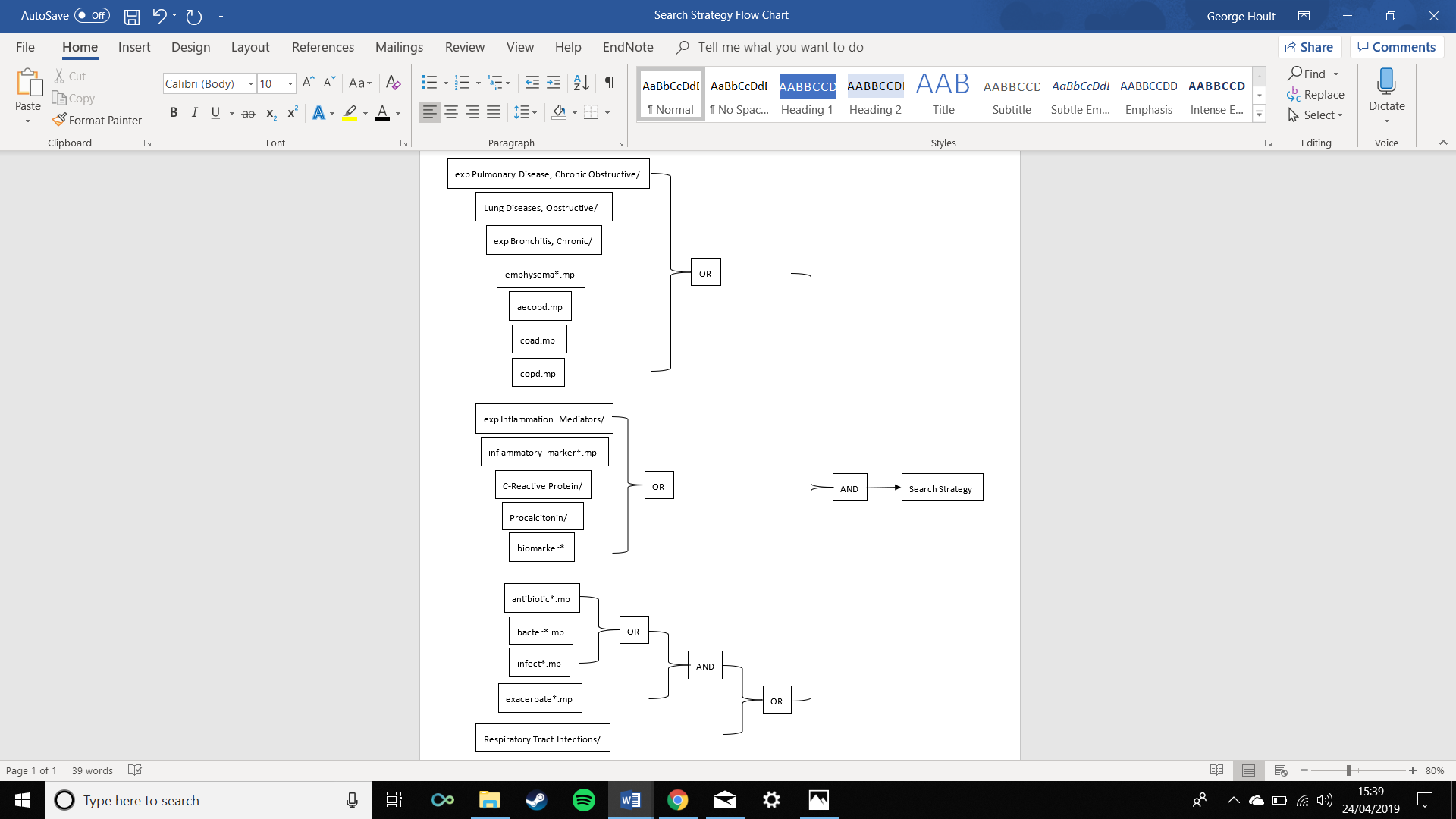
**

Supplement: Supplementary file 1 — Additional file 1. Supplementary Figure S1. Search strategy. [file 12890_2022_1958_MOESM1_ESM.docx]
